# Whole-cell tumor vaccines desialylated to uncover tumor antigenic Gal/GalNAc epitopes elicit anti-tumor immunity

**DOI:** 10.1186/s12967-022-03714-y

**Published:** 2022-10-31

**Authors:** Jianmei Huang, Meiying Li, Bingjie Mei, Junyang Li, Yi Zhu, Qiaoshan Guo, Jianming Huang, Guonan Zhang

**Affiliations:** 1 School of Medicine, University of Electronic Science and Technology of China, Chengdu, China; 2 Biochemistry and Molecular Biology, Sichuan Cancer Institute, Chengdu, China; 3 Department of Ultrasound, Sichuan Cancer Hospital, School of Medicine, University of Electronic Science and Technology of China, Chengdu, China; 4 Department of Gynecologic Oncology, Sichuan Cancer Hospital, School of Medicine, University of Electronic Science and Technology of China, Chengdu, China

**Keywords:** Sialoglycans, Gal/GalNAc, Siglec-9, MGL, Tumor vaccine, TCR-Vβ repertoire

## Abstract

**Background:**

Aberrant sialoglycans on the surface of tumor cells shield potential tumor antigen epitopes, escape recognition, and suppress activation of immunocytes. α2,3/α2,6Gal- and α2,6GalNAc (Gal/GalNAc)-linked sialic acid residues of sialoglycans could affect macrophage galactose-type lectins (MGL) mediated-antigen uptake and presentation and promote sialic acid-binding immunoglobulin-like lectins (Siglecs) mediated-immunosuppression. Desialylating sialoglycans on tumor cells could present tumor antigens with Gal/GalNAc residues and overcome glyco-immune checkpoints. Thus, we explored whether vaccination with desialylated whole-cell tumor vaccines (DWCTVs) triggers anti-tumor immunity in ovarian cancer (OC).

**Methods:**

Sialic acid (Sia) and Gal/GalNAc residues on OC A2780, OVCAR3, and ID8 cells treated with α2-3 neuraminidase (α2-3NA) and α2-6NA, and Sigec-9 or Siglec-E and MGL on DCs pulsed with desialylated OC cells were identified using flow cytometry (FCM); RT-qPCR determined IFNG expression of T cells, TRBV was sequenced using Sanger sequencing and cytotoxicity of αβ T cells was measured with LDH assay; Anti-tumor immunity in vivo was validated via vaccination with desialylated whole-cell ID8 vaccine (ID8 DWCTVs).

**Results:**

Gal/GalNAc but not Sia residues were significantly increased in the desialylated OC cells. α2-3NA-modified DWCTV increased MGL but decreased Siglec-9 or Siglec E expression on DCs. MGL^bright^/Siglec-9^dim^ DCs significantly up-regulated *IFNG* expression and CD4/CD8 ratio of T cells and diversified the TCR repertoire of αβ T-cells that showed enhanced cytotoxic activity. Vaccination with α2-3NA-modified ID8 DWCTVs increased MGL^bright^/Siglec-E^dim^ DCs in draining lymph nodes, limited tumor growth, and extended survival in tumor-challenged mice.

**Conclusion:**

Desialylated tumor cell vaccine could promote anti-tumor immunity and provide a strategy for OC immunotherapy in a clinical setting.

**Supplementary Information:**

The online version contains supplementary material available at 10.1186/s12967-022-03714-y.

## Background

Cell surface glycans on membrane-bound proteins are essential regulators of the immune system that regulate immune cell development and function, modulate immune receptor interactions, and serve as ligands for glycan-binding proteins (lectins) expressed by immune cells [1, 2]. Terminal sialic acid residues are abundant in glycan chains of glycoproteins and glycolipids on the surface of all live cells forming an outer layer of the cell known initially as glycocalyx [3]. Moreover, they mediate several of the regulatory functions of glycans in the immune system. Sialic acid-containing glycans (sialoglycans) are involved in numerous molecular interactions at the cell surface and have several direct and indirect functions in the immune system [4, 5].

Tumor antigens produced by somatic DNA mutations due to the inherent genetic instability are glycosidically hypersialylated. Aberrant glycosylation is a common feature of many cancers. Alteration of cell surface sialylation is seen with transformation to a tumor cell. Altered glycosylation sites in epithelial cancer cells may play an essential role in tumor progression, as they may affect tumor cell migration and antigen presentation by antigen-presenting cells (APCs) [6–8]. Altered sialylation has long been associated with the malignancy of carcinoma. High expression of sialic acids has been proposed to protect cancer cells from recognition and eradication by the immune system [9].

It has been demonstrated that sialic acids linked to galactose (Gal) and *N*-acetylgalactosamine (GalNAc) via a-2,3- and a-2,6-linkages significantly increased in cancer compared to normal tissues [10, 11]. They allow tumor cells to become 'invisible' to avoid immune recognition by DCs as APCs and attack by anti-tumor T cells. The macrophage galactose-type lectin (MGL) on DCs has been shown to induce immunosuppressive responses upon recognizing aberrant sialylation on cancer cells [12, 13]. MGL is the lectin that exclusively binds terminal Gal/GalNAc epitopes of tumor-associated glycan [14, 15]. It is an antigen-uptake receptor for the internalization of Gal/GalNAc carrying immunogens delivered into MHC class I and II compartments, thus improving DC performance to facilitate MHC-restricted antigen presentation to T cells and, most importantly, increasing antigen-specific CD8 + T-cell activation [16–19].

Sialoglycans are recognized by sialic acid-specific receptors on immune cells, such as sialic acid-binding immunoglobulin-like lectins (Siglecs). The human Siglec family consists of 15 members that are broadly expressed by the majority of immune cells. They can be categorized as structurally conserved Siglecs (Siglec-1, -2, -4, and -15) and CD33-related Siglecs (CD33 or Siglec-3, Siglec-5, -6, -7, -8, -9, -10, -11, -12, -14, and -16). As numerous human malignancies express ligands for both Siglec-7 and Siglec-9, the CD33-related Siglec-7 and Siglec-9 are of special importance in the context of tumor immunotherapy [20, 21]. Siglec-7 and Siglec-9 are reported to influence NK cell-dependent tumor immunosurveillance [20]. Siglec-7 binds to 2,8-sialyl residues with the highest affinity, while Siglec-9 preferentially binds to 2,6-sialyl and 2,3-sialyl residues [22]. The sialoglycan-Siglec interaction suppresses an immune response as sialic acids are considered self-associated molecular patterns [20, 23–25]. The surface glycan profile of human tumor cells was dominated by α-2,3- and α-2,6-linked sialic acid-capped complex *N*-glycans and bi-antennary N-glycans. Sialic acids can trigger immune inhibitory Siglecs on DCs, affecting antigen presentation to CD4 + and CD8 + T cells [26]. Gal/GalNAc-linked sialic acids of sialoglycans are known to interact with Siglec 9, an inhibitory immune checkpoint expressed broadly on immune cells, including DCs, which can impede T cell-mediated anti-tumor responses through immunoreceptor tyrosine-based inhibitory motifs (ITIM) [27–31].

Poor presentation of tumor antigens to the immune system remains a major obstacle to effective anti-tumor vaccine therapy [32]. Aberrant sialylation of tumor antigens enhances immune evasion and reduces the efficacy of several types of available immunotherapies, including tumor vaccine therapy [9, 33].

These findings suggest that removing α-2,3Gal- and α-2,6GalNAc-linked sialic acid residues from sialoglycans on the surface of tumor cells could improve tumor antigen presentation by DCs, block Siglec-sialic acid interactions, and boost anti-tumor immune responses. Thus, targeted desialylation of sialoglycan on tumor cells can be used to design novel anti-tumor vaccines [29, 34–36]. Here, we selectively removed the α-2,3- and α-2,6-linked sialic acids on OC cells by α2-3 and α2-6 neuraminidases, respectively, to generate desialylated whole-cell tumor vaccines (DWCTVs), resulting in the novel delivery approach for tumor antigens with Gal/GalNAc epitopes. Increased Gal/GalNAc epitopes accompanied the decrease of sialic acids on OC cells. DWCTVs could mature and activate DCs, reduce Siglec-9 and elevate MGL on DCs. These Sigec-9^dim^/MGL^bright^ DCs diversified the TCR-Vβ repertoires of T cells, resulting in enhanced cytotoxicity against the parental OC cells in vitro. Vaccination with DWCTVs could delay tumor formation, limit tumor growth, and prolong the survival of mice inoculated with tumor cells. The "win–win" effects of DWCTVs might be their blocking of Siglec-sialic acid interaction and increasing MGL-Gal/GalNAc interaction between immune cells and OC cells. DWCTVs in our study provided a promising therapeutic option to render tumors permissive to immune attack to reduce or prevent tumor recurrence.

## Methods

### Cell lines and animals

Human OC cell lines (OVCAR-3 and A2780) and the mouse OC cell line (ID8) were purchased from the Committee on Type Culture Collection of the Chinese Academy of Sciences. OVCAR-3 cells were maintained in McCoy's 5A (Sigma), containing1% human insulin and 15% fetal calf serum (FCS). A2780 and ID8 cells were cultured with RPMI-1640 (Gibco) and DMEM (Gibco), respectively, supplemented with 10% FCS. Penicillin (100 IU mL^−1^) and gentamicin (40 IU mL^−1^) were added to the mediums for these three cells. All cells were cultured in a humidified incubator at 37 °C with 5% CO_2_.

Specific pathogen-free, 6–8-week-old female C57BL/6 littermate mice (20 ± 2 g) (Dossy Experimental Animals Co. LTD Chengdu, China) were used for tumorigenicity studies. Animal studies were carried out following protocols approved by the Ethics Committee of Sichuan Cancer Hospital (SCCHEC-04-2019-004).

### Preparation and characterization of DWCTVs

The cells (1 × 10^6^ cells/mL) were pre-treated with 100 ng mL^−1^ of mitomycin C (R&D, 3258/10) in serum-free medium for 3 h at 37 °C, washed and resuspended, and then desialylated with 100 mU mL^−1^ of α2-3 and α2-6 neuraminidases (NAs) from *Clostridium perfringens* (Sigma, N2876) and *Arthrobacter ureafaciens* (Roche, 10269611001) in the same medium for 30 min at 37 °C, respectively. The terminal α2,3/α2,6 sialic acid and Gal/GalNAc residues on the resultant cells were identified using flow cytometry (FCM) with Biotinylated MALII/DyLight 488 streptavidin (B-1265-1/SA-5488-1), Fluorescein labeled SNA (FL-1301), and Cy5 labeled PNA (CL-1075-1).

### DC cultures and maturation

Peripheral blood mononuclear cells (PBMCs) were isolated from the healthy adult female volunteer by Ficoll-Hypaque (1.077 g mL^−1^) gradient centrifugation and plated in a complete RPMI-1640 medium in culture plates for 4 h at 37 °C. The adherent monocytes were cultured in a complete RPMI-1640 medium with 500 U mL^−1^ interleukin 4 (IL 4) (R&D, 204-IL-010) and 150 ng mL^−1^ granulocyte–macrophage colony-stimulating factor (GM-CSF) (R&D, 215-GM-010) for 7 consecutive days. After then, the immature DCs (imDCs) were harvested, washed, and pulsed with an equal number of WCTV cells in the same medium containing 250 U mL^−1^ IL 4 and 75 ng mL^−1^ GM-CSF for 3 days. The mature DCs (mDCs) were then analyzed by flow cytometry for DC-surface maturation and activation markers (CD86, CD83), Siglec-9, and MGL expression.

### T cell stimulation with the mDCs

The nonadherent cells of PBMCs were harvested, washed twice with PBS, and then incubated with the mDCs at a 10:1 ratio of cell number overnight. The stimulated lymphocytes were harvested to detect *IFNG* expression by RT-qPCR and CD4/CD8 ratio by FCM. Then these lymphocytes were expanded in the same medium with 500 U mL^−1^ IL-2 (Solarbio, GMP-11848-HNAE) for 5 days. The TCR-Vβ sequences and cytotoxicity of the resultant T cells were identified by Sanger sequencing and LDH assay, respectively.

### Real-time quantitative PCR for *IFNG* expression of human T cells

Total RNA was extracted from human T cells using TRIzol reagent (Invitrogen, 15596026). A total of 2 μg RNA was reverse transcribed using Eastep RT Master Mix Kit (Promega, LS2052), and 1 μL cDNA was used for qPCR using Eastep qPCR Master Mix Kit (Promega, LS2062), and the primer pairs for human *IFNG*: forward: 5′-TCGGTAACTGACTTGAATGTCCA-3′, reverse: 5′-TCGCTTCCCTGTTTTAGCTGC-3′ and the primer pairs for human *GAPDH*: forward 5′-CAAGGTCATCCATGACAACTTTG-3, reverse 5′-TCGCTTCCCTGTTTTAGCTGC-3′

### FCM assay

All lectins and antibodies were obtained directly from Vector Labs or BD Pharmingen, R&D, Biolegend, Abcam, and Servicebio. According to the manufacturers' protocols, cells were incubated with antibodies for 30 min, and analysis was performed on a BD FACS Canto II flow cytometer.

### TCR-Vβ repertoire sequencing

TCR beta-chain variable (*TRBV*) gene of human T cells was PCR-sequenced. Briefly, to PCR-amplify and clone the sequences of TCR β-chains, 5′RACE was performed with 1.0 μg of total RNA from human T cells co-incubated with mDCs using RLM-RACE Kit (Thermo, AM1700) and primers specific for the gene (inner primer 5′-TTCTGATGGCTCAAACAC-3′ and outer primer 5′-GGTCCACTCGTCATTCTCCGA-3′). Then, 4 μL of the recovered and purified DNA fragments larger than 400 bp were ligated into 1 μL pEASY T3 cloning vector (TRANS, CT301). The cloned DNA was sequenced using Sanger sequencing and analyzed using the IMGT/V-QUEST online tool (IMGT-the international ImMunoGeneTics information system, http://www.imgt.org) [37, 38]. The entire full length of TRBV is defined as a cluster containing all three complementarity determining regions (CDRs).

### Cytotoxicity assay

Cytotoxicity of T cells was measured by the LDH assay kit (Promega, CytoTox 96^®^-G1780). Briefly, T cells and OC cells were mixed by the ratio of effect and target at 1:1, 5:1, and10:1 in 1% FCS RPMI 1640 medium in a 96-well V-bottom for 4 h at 37 °C. The subsequent operations were performed according to the manufacturer's procedure. The percentage of cytotoxicity is calculated with the following formula, % cytotoxicity = (experimental-effector spontaneous-target spontaneous)/(target maximum-target spontaneous) × 100.

### IL-2 and IFNγ ELISA

Mice were vaccinated with whole-cell ID8 vaccines, and the serum was frozen at − 80 °C. IL-2 and IFNγ in serum were quantified using mouse IL-2 and IFNγ ELISA plates (R&D VAL607 and VAL602). Plates were analyzed with an Infinite Tecan M200 PRO microplate reader (Switzerland).

### Mouse T cell subsets and DCs assay

Single-cell suspensions from tumor-draining lymph nodes of the vaccinated mice with whole-cell ID8 vaccines were prepared. *FCM* analysis of T cell subsets and DCs were performed with antibodies specific for CD3e (BD, 553061), CD4 (BD, 553051), CD8a (BD, 552877), and antibodies specific for Siglec E (R&D, FAB5806A), MGL1/2 (R&D, FAB4297P), CD11c (Biolegend, 117324), CD86 (Biolegend, 105116).

### Fluorescent microscopy

Mice ID8 tumor tissue sections with antigen repair were immunostained with primary antibodies against murine CD8 and CD39, and FITC and Cy3 fluorescein-conjugated secondary antibodies against primary antibodies for imaging with fluorescent microscopy and analysis of the intensity of staining with Image J (Fiji) software.

### Tumorigenicity studies

Animals were randomly grouped, and all animals were included in the data analysis except those who unexpectedly died. Female mice were inoculated subcutaneously (s.c.) once a week for three consecutive weeks at one side axilla with 2 × 10^5^ whole-cell ID8 vaccines prepared with ID8 tumor tissue as stated above or PBS before subcutaneous challenge with 1 × 10^6^ parental ID8 cells in 100 μL PBS at the opposite axilla. Tumor volume at the opposite axilla was determined using calipers. Mice were sacrificed when tumor diameter exceeded 1 cm. The tumor growth and survival outcome were used to evaluate the efficiency of desialylated whole-cell ID8 vaccines in inducing specific anti-tumor immunity.

### Statistical analysis

All statistical analyses were performed using GraphPad Prism 8. Statistical analysis was carried out using a paired t-test, unpaired t-test, and the log-rank (Mantel-Cox) test. A P-value of less than 0.05 was considered statistically significant.

## Results

### Characterization of DWCTVs with Gal/GalNAc epitopes

**Fig. 1:**
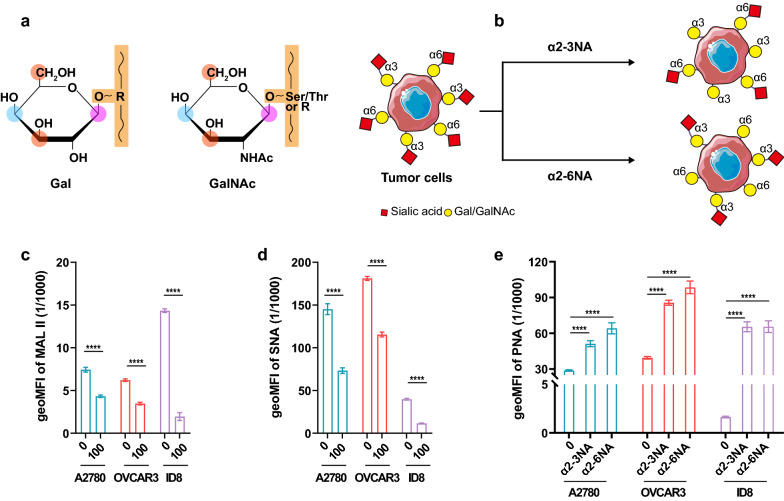
Preparation and Characterization of DWCTVs. **a** Schematic illustration of the generation of desialylated whole-cell tumor vaccines (DWCTVs) using NAs. Terminal sialic acids of sialoglycans on OC cells were selectively cleaved by α2-3NA and α2-6NA, and the underlying Gal/GalNAc were exposed. **b** α-2,3-linked sialic acids on DWCTVs were reduced via 100 mU mL^−1^ α2-3NA modification. The geoMFI of MAL II bound to α-2,3-linked sialic acids were shown. **c** α-2,6-linked sialic acids on DWCTVs were reduced via 100 mU mL^−1^ α2-6NA modification. The geoMFI of SNA bound to α-2,6-linked sialic acids were shown. **d** Gal/GalNAc epitopes on DWCTVs were increased via both α2-3NA and α2-6NA modifications. The geoMFI of PNA bound to Gal/GalNAc residues was shown. **e** Chemical strructure of Gal and GalNAc. R groups reprent monosaccharides, such as Gal, GalNac, Glc and GlcNAc. The 1-hydroxyl (purple) of GalNAc could attach to Ser/Thr. Sialic acids usually link to the 3- or 6-hydroxyl (red) groups of Gal/GalNAc. The 1-, 3- and 4-hydroxyl (blue) groups of them usually contribute to the glycan elongation. 3- and 4-hydroxyl groups of them are essential for Ca2 + binding. Data are presented as mean ± SD. The statistical test of **b–d** used is the unpaired t-test. ****P < 0.0001

There are four hydroxyl groups on Gal and GalNAc that may form glycosidic bonds (Fig. 1a). 1-hydroxyl of GalNAc is attached to the hydroxyl group of the protein serine (Ser) or threonine (Thr) residues for initiation of glycosylation, and 3- and 6-hydroxyls of GalNAc could be linked with sialic acids to terminate glycosylation. To uncover Gal/GalNAc epitopes on OC cells, we desialylated sialic acids on intact cells by hydrolysis with α2-3NA and α2-6NA following mitomycin C to generate DWCTVs (Fig. 1b; Additional file 1: Fig. S1, Additional file 2: Fig. S2).

FCM showed that there was a significant decrease of α-2,3Sia and α-2,6 Sia residues and an increase of Gal/GalNAc epitopes on the surface of DWCTVs compared to the corresponding controls (*P* < 0.0001) (Fig. 1c–e; Additional file 3: Fig. S3).

### Maturation and activation of DCs induced by DWCTVs in vitro

**Fig. 2:**
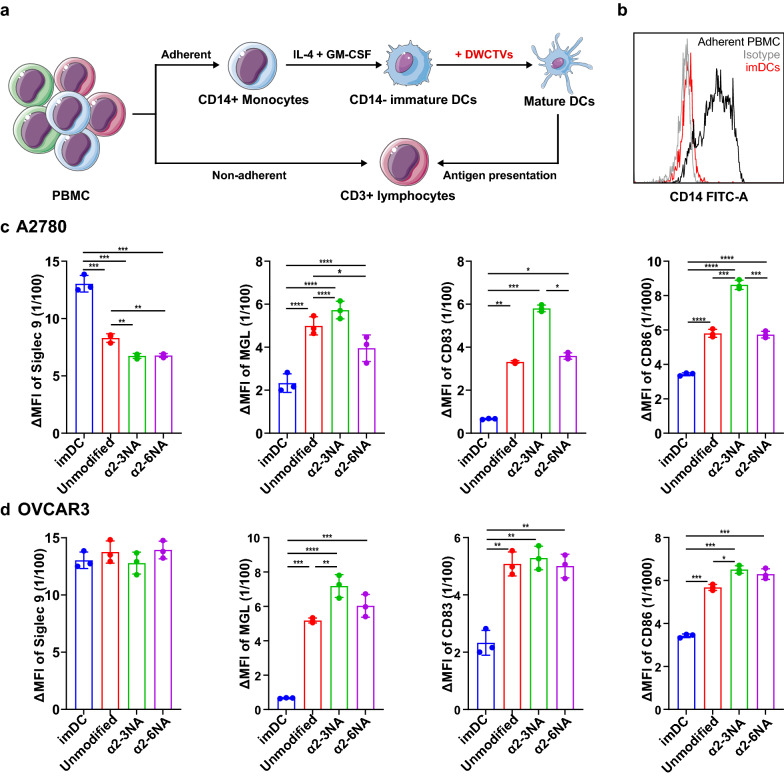
DC maturation and activation induced by DWCTVs in vitro. **a** The schematic illustration of in vitro immune cell stimulation assays. **b** The detection of CD14 expression on adherent PBMCs and imDCs by FCM. PBMCs were differentiated into CD14- imDCs by IL 4 and GM-CSF stimulation. **c**, **d** Quantification of Siglec 9, MGL, CD83, and CD86 expression on the surface of DCs after stimulation by A2780 and OVCAR3 DWCTVs. The Siglec 9, MGL, CD83, and CD86 were detected by FCM and analyzed by MFI. ΔMFI = MFI of test tube—MFI of the unstained tube. Unmodified, α2-3NA and α2-6NA represent the stimulation of unmodified WCTV, α2-3NA modified DWCTV and α2-6NA modified DWCTV, respectively. Data are presented as mean ± SD (*n* = 3). The statistical test for (**c**, **d**) used is the unpaired t-test. *P < 0.05; **P < 0.001; ***P < 0.005; ****P < 0.0001

To explore the impact of α-2,3Sia and α-2,6Sia shedding on DC performance, we co-cultured immature DCs (imDCs) with DWCTVs (Fig. 2a). As shown in Fig. 2b–d, DCs (CD14-imDCs) from monocytes of the healthy female were pulsed with DWCTVs (Additional file 4: Fig. S4) derived from A2780 but not OVCAR3 cells exhibited that the expression of differentiation marker CD83, co-stimulatory molecule CD86, and MGL (*P* < 0.05) were remarkably increased, but Siglec-9 was decreased (*P* < 0.01), as compared to the corresponding controls. Interestingly, the phenotypic alterations of DCs stimulated with α2-3NA modified DWCTVs were more obvious than those with α2-6NA modified ones, suggesting that the removal of α-2,3-linked sialic acids on the terminal galactosyl of GalNAc may play an important role for activation and performance of DCs.

### DCs loaded with DWCTVs activate T cells in vitro

**Fig. 3:**
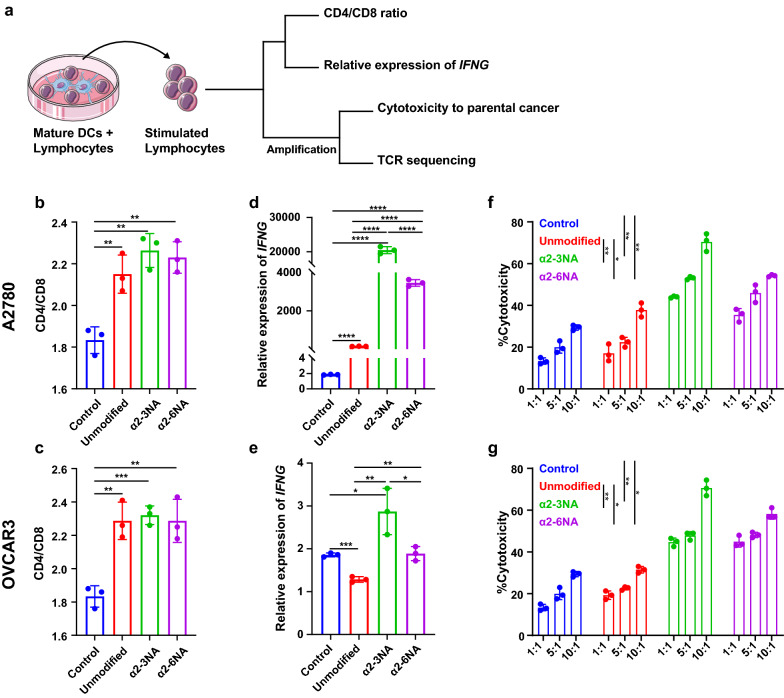
Lymphocytes stimulated by DCs pulsed with DWCTVs in vitro. **a** Flow chart of experimental plan adopted for the in vitro lymphocyte stimulation assays. **b**, **c** The CD4/CD8 ratio of CD3 + cells. **d**, **e** *IFNG* mRNA expression level of lymphocytes. The *IFNG* mRNA expression level of lymphocytes stimulated by imDC and DCs pulsed with A2780 and OVCAR3 DWCTVs was detected by RT-qPCR. The relative expression level was normalized to the *GAPDH* expression level and calculated by 2^−ΔCT^ × 1000. **f**, **g** DWCTVs enhance DC-mediated cytotoxicity of T cells in *vitro*. The killing activity of corresponding T cells (effector cells) to parental OC cells (target cells) shown are from the effector/target ratio at 1:1, 5:1, and 10:1. Unmodified, α2-3NA and α2-6NA represent the stimulation of DCs pulsed with unmodified WCTV, α2-3NA modified DWCTV and α2-6NA modified DWCTV, respectively. Data are presented as mean ± SD (*n* = 3). The statistical test of **b–e** used is an unpaired t-test, while it is paired t-test for (**f**–**g**). *P < 0.05; **P < 0.01; ***P < 0.005; ****P < 0.0001

To test whether DCs loaded with DWCTVs could activate T cells, the CD4/CD8 ratio and *IFNG* expression of T cells were detected following exposure of PBLs to DCs loaded with DWCTVs (Fig. 3a). The results showed that CD4/CD8 ratio and *IFNG* expression in CD3 + T cells were markedly increased, as compared to the controls (*P* < 0.05), and that DCs loaded with α2-3NA modified DWCTVs could prime activation of T cells more effectively than α2-6NA modified DWCTVs (Fig. 3b–e).

### DCs loaded with DWCTVs change TCR-Vβ repertoire

To prove the immunogenicity of DCs loaded with DWCTVs, we examined whether these DCs modulate the TCR-Vβ repertoire diversity of αβ T cells. As shown in Table 1, *TRBV* gene sequencing identified no difference in *TRBV* sequences between the control T cells and T cells co-incubated with imDCs. The *TRBV* sequences of T cells co-incubated with DCs pulsed with primary whole-cell tumor vaccines derived from A2780 (9.52% of CDR1/CDR2 AA sequences) and OVCAR3 cells (85.71% of CDR1/CDR2/CDR3 AA sequences) were partially identical to that of the control T cells. Interestingly, there was a great diversity in *TRBV* sequences between T cells stimulated with DCs loaded with DWCTVs and the control T cells. The T cells stimulated with DCs loaded with DWCTV derived from A2780 and OVCAR3 cells fell into 12 and 6 distinct *TRBV* sequences, respectively (Table 1). These demonstrated that the immunogenicity of DCs conferred by DWCTVs could modulate the TCR-Vβ repertoire diversity of αβ T cells.

**Table 1:** The TCR-Vβ repertoires of αβ T cells

Spec	V_gene	J_gene	D_gene	CDR1	CDR2	CDR3	Freq (%)
Control	TRBV30	TRBJ2-5	TRBD2	GTSNPN	SVGIG	AWRTGAGQY	100.0
imDC	TRBV30	TRBJ2-5	TRBD2	GTSNPN	SVGIG	AWRTGAGQY	100.0
Unmodified	TRBV30	TRBJ1-5	TRBD1	GTSNPN	SVGIG	AWSHGNNQPQH	4.76
(A2780)	TRBV30	TRVJ2-2	TRBD2	GTSNPN	SVGIG	AWSNWRGGPGELF	4.76
	TRBV5-1	TRBJ2-7	TRBD1	SGHRS	YFSETQ	ASSLRLGDEQY	23.81
	TRBV6-6	TRBJ1-1	TRBD2	MNHNY	SVGAGI	ASSYEGGPGTEAF	14.29
	TRBV20-1	TRBJ1-4	TRBD1	DFQATT	SNEGSKA	SASGLLEEKLF	52.38
α2-3 NA	TRBV24-1	TRBJ1-2	-	KGHDR	SFDVKD	ATSYT	5.00
(A2780)	TRBV20-1	TRBJ2-3	TRBD1	SLARSH	SNEGSKA	SANTGSTDTQY	5.00
	TRBV7-2	TRBJ2-3	TRBD2	SGHTA	FQGNSA	ASSSRSRLAGAEDTQY	5.00
	TRBV28	TRBJ2-7	TRBD1	MDHEN	SYDVKM	ASSHRYEQY	5.00
	TRBV10-1	TRBJ2-7	TRBD2	CVCW	SYGVQD	ASCSGSDPILRAV	10.00
	TRBV11-3	TRBJ2-5	TRBD2	SGHNT	YENEEA	ASSPQLAGVFKTQY	10.00
	TRBV28	TRBJ1-2	TRBD1	MDHEN	SYDVKM	ASTAGSNYGYT	10.00
	TRBV2	TRBJ1-5	TRBD2	SNHLY	FYNNEI	ASSAPLSNQPQH	50.00
α2-6 NA	TRBV29-1	TRBJ2-2	TRBD1	SQVTM	ANQGSEA	SAVLRGTGELF	14.29
(A2780)	TRBV6-6	TRBJ2-1	TRBD2	MNHNY	SVGAGI	ASRKAEGIYNEQF	19.04
	TRBV20-1	TRBJ1-5	TRBD1	DFQATT	SNEGSKA	SARVQANQPQH	23.81
	TRBV7-8	TRBJ1-5	TRBD1	SGHVS	FQNEAQ	ASSAGWGPQH	42.86
Unmodified	TRBV27	TRBJ1-1	TRBD2	MNHEY	SMNVEV	ASSFQRGTEAF	14.29
(OVCAR3)	TRBV30	TRBJ2-5	TRBD2	GTSNPN	SVGIG	AWRTGAGQY	85.71
α2-3 NA	TRBV6-2	TRBJ2-1	TRBD1	MNHEY	SVGEGT	ASSPTSPRTPEQF	50.00
(OVCAR3)	TRBV5-6	TRBJ2-5	TRBD2	SGHDT	YYEEEE	ASAPGGMHEQY	50.00
α2-6 NA	TRBV3-1	TRBJ2-6	TRBD1	LGHDT	YNNKEL	ASSLLGYSGANVLT	9.52
(OVCAR3)	TRBV21-1	TRBJ1-5	TRBD1	TISF*	SQNEEL	ASSKIRTGVAISPS	14.29
	TRBV29-1	TRBJ2-6	TRBD2	SQVTM	ANQGSEA	SAAASGANVLT	23.81
	TRBV2	TRBJ2-1	TRBD1	SNHLY	FYNNEI	ASSDQSGQGEF	52.38

TCR β-chains of control T cells and T cells stimulated by imDC and NA modified/unmodified A2780/OVCAR3 cells antigen-loaded DCs were determined by TRBV gene sequencing. Each row represents one clone, and its frequency (Freq.) in its group is indicated to the right. *Stop codon, Spec: specimen

### DWCTVs enhance DC-mediated cytotoxicity of T cells in *vitro*

To further prove the anti-tumor activity of αβ T cells with the diversified TCR-Vβ repertoires by the DCs loaded with DWCTVs, we test the cytotoxicity of the activated αβ T cells on A2780 and OVCAR3 cells. The LDH assay showed that the killing efficiencies of these T cells significantly improved as compared to the corresponding controls (*P* < 0.05) (Fig. 3f, g). Together, our findings showed that DCs pulsed with DWCTVs not only induced the activation of T cells with high-affinity tumor antigen-specific TCRs but also enhanced their killing activity against parent tumor cells.

### Activation of immune cells by DWCTVs in vivo

**Fig. 4:**
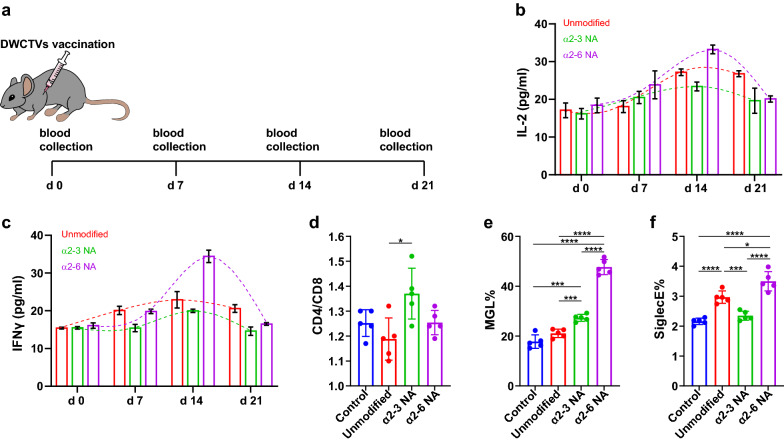
Immune responses elicited by DWCTVs in vivo. **a** Timeline of vaccination and sample collection. **b**, **c** IL-2 and IFNγ plasma levels of vaccinated mice were monitored and detected by ELISA. **C** The CD4/CD8 ratio of CD3 + T cells in DLNs at the 14th d after vaccination. **d**, **e** Quantification of Siglec E and MGL expression on DCs located in DLNs at the 14th d after vaccination. The Siglec-9 + and MGL + DCs are gated on CD11c + CD86 + double-positive cells. Data are presented as mean ± SD (*n* = 5). Each plot of **d–f** represents the mean of three technical replicates of a mouse. The statistical test of **d–f** used is an unpaired t-test. *P < 0.05; ***P < 0.005; ****P < 0.0001

To verify whether DWCTVs can elicit activation of immune cells in *vivo*, female mice were vaccinated with the inactivated DWCTVs derived from ID8 cells to test the plasma IFNγ and IL-2, T cell subtypes, and MGL and Siglec E of DCs in draining lymph nodes (DLNs) (Fig. 4a). Our results showed that the plasma levels of IFNγ and IL-2 reached the peak, and the CD4/CD8 ratio of T cells in DLNs increased from 1.25 ± 0.05 to 1.37 ± 0.10 (*P* < 0.05) (Fig. 4d) around 14 days after the vaccination. Meanwhile, vaccination with α2-3NA modified ID8 DWCTV induced an increased expression of MGL, not Siglec E of CD11c + /CD86 + DCs, while vaccination with α2-6NA modified ID8 DWCTV induced an increased expression of both MGL and Siglec E; however, inoculation with primary ID8 WCTVs induced an increased expression of Siglec E but not MGL (Fig. 4e, f). These findings suggest that vaccination with DWCTVs rather than primary WCTV could evidently elicit immune activation in *vivo*.

### Vaccination with DWCTVs limits tumor growth and prolongs survival of mice bearing tumor

**Fig. 5:**
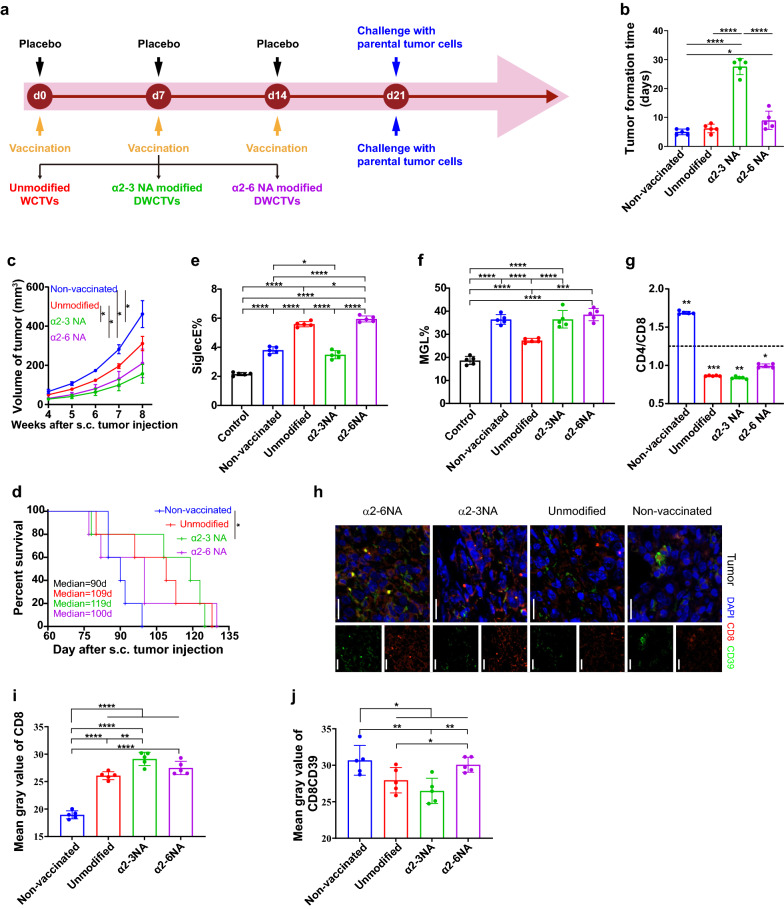
Effects of DWCTVs on tumorigenicity studies. **a** The timeline of the in *vivo* tumor challenge experiment. **b** The tumor formation time after tumor challenge. **c** The tumor growth curve of tumor-bearing mice. **d** The survival curves of tumor-challenged mice. **e**, **f** Quantification of Siglec E and MGL expression on DCs located in DLNs at the 7th d after tumor injection. **g** CD4/CD8 ratio of T cells in DLNs at the 7th d after tumor inoculation. **h** Representative dual immunofluorescence images for TILs in tumor tissues after mice were sacrificed at 50th d. Red, CD8; Green, CD39; Scale bar 20 μm. **i** The Mean gray value of CD8 pixel for the tumor section. **j** The Mean gray value of CD8 and CD39 double-positive pixel for the tumor section. The control mice represent normal mice without vaccination and tumor injection. Unmodified, α2-3NA and α2-6NA represent the vaccination with unmodified WCTV, α2-3NA modified DWCTV, and α2-6NA modified DWCTV, respectively. Each plot of **e–g** represents the mean of three technical replicates of a mouse. Each plot of **i**, **j** represents the average of 3 fields of view per tumor section. The statistical test of (**e–g**, **i**, **j**) used is unpaired t-tests, while it is paired t-test for (**c**). The survival curve of (**d**) is analyzed using the log-rank test. *P < 0.05; **P < 0.01; ***P < 0.001; ****P < 0.0001

To determine whether ID8 DWCTVs elicit anti-tumor immunity in *vivo*, tumorgenicity of parental ID8 cells, tumor growth, and survival of mice bearing ID8 tumor were evaluated after vaccination with ID8 DWCTVs once a week for 3 weeks in challenging mouse tumor models (Fig. 5a). Vaccinations with α2-3NA and α2-6NA modified ID8 DWCTVs significantly delayed tumor formation time (27.60 ± 2.79 days and 9.00 ± 3.16 days, respectively) compared to the parental ID8 cells and primary ID8 WCTV controls (5.00 ± 1.00 days and 6.20 ± 1.48 days, respectively, both *P* < 0.05) (Fig. 5b; Additional file 5: Fig. S5), also clearly limited the tumor growth (Fig. 5c). Interestingly, vaccination with α2-3NA modified ID8 DWCTVs had more significant effects than α2-6NA modified ID8 DWCTV in postponing tumor progression (Fig. 5c). Furthermore, vaccination with α2-3NA, not α2-6NA modified ID8 DWCTV notably prolonged overall survival of mice bearing ID8 tumor (median OS 119 days, *n* = 5) compared to the primary WCTV control (median OS 90 days, *n* = 5) (Fig. 5d) (*P* < 0.05), suggesting that α2-3NA modified DWCTV could prime the anti-tumor immunity against OC.

**Fig. 6:**
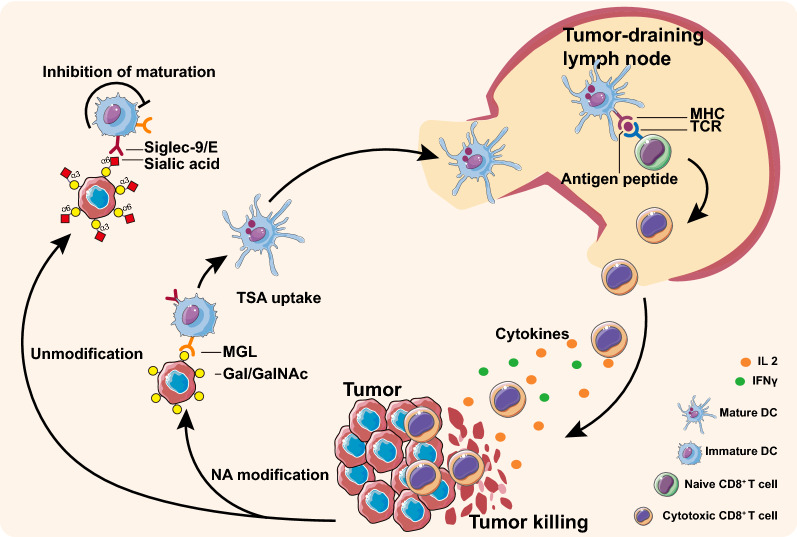
Schematic diagram showing possible actions of DWCTVs

Moreover, CD8 + T cells and MGL not Siglec E of DCs from DLNs significantly increased in vaccinated mice inoculated with parental OC cells for 7 days (Fig. 5e–g), suggesting that ID8 DWCTVs could be able to induce the proliferation and activation of memory CD8 + cells. Immunofluorescence histochemistry (IFHC) revealed that vaccination with α2-3NA modified ID8 DWCTV significantly increased effector CD8 + T cells and decreased CD8 + /CD39 + exhausted T cells in tumor-infiltrating lymphocytes (TILs) of ID8 tumor tissues compared to the corresponding controls (Fig. 5h–j), suggesting that DWCTVs could also contribute to the anti-tumor immune effect on the local tumor. These might indicate that DWCTVs could activate immune cells in the draining lymph node, and these immune cells circulate to tumor tissues and exert anti-tumor effects (Fig. 6).

## Discussion

Tumor-derived sialoglycans can target different aspects of the immune system to promote evasion responses [39]. Tumor cells mask their specific antigens by glycan modification which is one of the molecular recognition patterns of immune evasion [34]. Moreover, the tumor-derived sialoglycans serve as potent immunomodulatory, forming an invisibility cloak shielding tumor cells from immune recognition [34, 39, 40]. The dense layer of sialoglycans on tumor cell surfaces avoids the normal occurrence of immunological synapses between cancer and immune cells [9, 41]. Such reduced recognition is believed to be enhanced by hypersialylation of tumor ligands for CLRs expressed by immune cells [42].

The aberrant sialylation forms complex glycocalyx lattices on the tumor cell surface, reducing tumor immunogenicity [39, 43, 44]. However, the underly Gal/GalNAc epitopes interact with MGL on DCs to trigger an immune response [12, 45]. Here, DWCTVs could expose tumor antigenic Gal/GalNAc epitopes and reduce sialic acids, conferring them with immunostimulatory potential. The 1-hydroxyl of GalNAc is attached to the hydroxyl group of the protein serine (Ser) or threonine (Thr) residues for initiation of glycosylation by *N*-acetylgalactosaminyltransferase (ppGalNAc-T) [46], and 3- and 6-hydroxyls of GalNAc could be linked with sialic acids to terminate glycosylation by α-2,3-sialyltransferase (ST3GAL ΙΙ) and α-2,6-sialyltransferase (ST6GAL I) [47]. Tn (GalNAcα1-O-Ser/Thr), T (Galβ1-3GalNAcα1-O-Ser/Thr), and STn (NeuAcα2-6GalNAcα1-O-Ser/Thr) antigens are widely expressed in several types of tumors [48]. Especially, the expression of STn on normal cells is limited but is abundant on OC, which makes STn a relatively specific tumor-associated antigen for vaccination [48]. ST3GAL ΙΙ transfers sialic acid to the 3-position of the distal GalNAc leading to tumor progression in OC [49–51]. Recently, the immunogenicity of GalNAc has paved its application in the targeted delivery of drugs [52, 53]. Our study's approach for DWCTVs production was desialylation of OC cells to expose Gal/GalNAc epitopes, which might have great potential to confer specific immunity to OC patients.

The glycan structures of tumor-associated sialoglycans on the surface of tumor cells could affect the interaction with MGL expressed on immature monocyte-derived DCs to orchestrate distinct immune responses. Aberrantly sialylated structures decorating cell surfaces on cancer cells have been shown to induce immunosuppressive responses of MGL on DCs [54]. In contrast, MGL-expressing APCs can take up Tn-derived peptide structures for antigen presentation and induction of T cell responses to elicit anti-tumor immunity [17, 55, 56]. Sialic acids on the termini of neighboring oligosaccharides significantly limit the peptide region recognized by APCs. The 3- and 4-hydroxyl groups of Gal/GalNAc are essential for Ca^2+^ binding required for MGL binding. The 2-acetamido group (NHAc) promotes the MGL binding, while the sialylation at 3-hydroxyl of GalNAc might interfere with MGL binding [16, 56], and exposure of the 3-hydroxyl group of GalNAc with α2-3NA treatment could enhance the affinity of binding MGL. The significantly increased DWCTV-DC interactions could result from the removal of steric hindrance formed by sialic acids and/or generation of (specific) interaction sites between uncapped galactosyl residues of GalNAc-Ser/Thr that could interact with MGL on the DCs [4].

Sialoglycans interact with Siglec-9 colocalized with the TCR-CD3 complex to inhibit TCR-mediated cell activation via recruitment of SHP-1 by ITIM phosphorylation to reduce ZAP 70 phosphorylation [57]. In addition, sialoglycans as alternative ligands of CD28 bind to CD80 on APCs, attenuating co-stimulatory signals of antigen-mediated activation of T cells [54]. Previous studies have found that aberrant sialic acid-Siglec interactions are associated with reduced anti-tumor immunity, which could be reversed by desialylation of tumor cells [29, 30]. Siglec-9 is an immunosuppressive sialic acid binding receptor and is involved in immunoregulation through ligation with glycoconjugates with terminal sialic acids on cancer cells. Studies showed that the expression of Siglec-9 was up-regulated in the development of monocytes into immature DCs and was decreased in mature DCs. Because Siglec-9 binds to both a2,3- and a2,6-sialic acid-linked glycoconjugates or sialoglycans, removal of terminal sialic acids from sialoglycans on the surface of cancer cells by α2-3 and α2-6NA or blocking of ligation with Siglec-9 by anti-Siglec-9 mAb could ameliorate maturation and activation of DCs [31, 58]. Furthermore, CD86, expressed on DCs, is an activating ligand of co-stimulatory receptor CD28 on T cells and is required for naiv̈e T cells to differentiate into functional effector cells [59]. Thus, by removing sialic acid ligands on the surface of tumor cells, CD28 is better able to engage CD80, accounting for increased co-stimulation of T cells [54]. As shown in our study, DWCTVs could expose tumor antigenic Gal/GalNAc epitopes masked by aberrant sialoglycans and facilitate Gal/GalNAc-MGL-mediated internalization and presentation of tumor antigens by mature DCs for activation of T cells.

The interaction of CD86^high^ DCs with T cells could induce the changes in T cell phenotypes and up-regulation of *IFNG* expression by CD8 + cytotoxic T cells (CTLs) in our study, indicating that DCs loaded with DWCTVs achieve immunogenic property and is capable of initiating T cell activation and proliferation. It is known that the two-signal model of T cell activation needs both peptide-MHC-TCR interaction and co-stimulatory CD28-CD80/CD86 interaction. The mature DCs with higher CD80/CD86 could induce co-stimulatory signals of antigen-mediated activation of naiv̈e T cells and a higher proportion of CD8 + CTLs [54].

As we know, conventional tumor vaccines target tumor-associated antigens (TAAs) overexpressed in cancers that are also expressed in normal tissues and can potentially induce central and peripheral tolerance responses, which results in low vaccination efficiency [60]. Moreover, the vaccines generated by intact tumor cells or tumor cell lysates also usually fail to elicit an effective immune response. Thus, DWCTV modified with α2-3NA could enhance the immunogenicity of DCs and provide co-stimulatory signals to activate T cells [61, 62]. CD8 + T cells are the critical effector cells that contribute to the anti-tumor immune response. They comprise various T-cell clones with diverse antigen-specific TCRs. Thus, elucidating the overall anti-tumor responses of diverse T-cell clones is an emerging challenge in tumor immunology. As we know, the lack of cellular immune response to tumor cells results from the poor presentation of antigens by DCs to CD8 + T cells and the inability of tumor vaccines to elicit sufficient DC activation to evoke the DC-derived co-stimulatory signals required to initiate effector CD8 + T-cell responses [63]. Fully effective tumor vaccines must elicit a diverse repertoire of CD8 + T-cell responses. The highly variable CDR3 of the TCR β chain is unique to individual T cell clones and can therefore be used to identify the T cell repertoire responses to the immunogenicity of DCs [64]. TCR-Vβ repertoire diversity of αβ T cells in our study demonstrates that DWCTVs could confer the immunogenicity of DCs. TCR diversity within antigen-specific T cell repertoires is essential for effective immunity to eliminate tumor cells [64]. The TCR repertoire of T cells is diversified at the initial phase and proliferated into the "best-fit" clonotypes by continuing exposure once they recognize the tumor antigens presented by the DCs. The enhanced killing activity here might indicate that the DCs could induce TCRs with optimal structural and affinity characteristics. Cancers with low TCR diversity are reported to be unable to recognize and eliminate tumor cells specifically [65]. Anti-tumor immune responses are highly individualized for the intrinsic differences in TCR repertoire contributed to the heterogeneity of anti-tumor immunity against the same tumors [66]. Together, our findings showed that DCs pulsed with DWCTVs induced the activation of T cells with high-affinity tumor antigen-specific TCRs and enhanced their killing activity against parent tumor cells.

Tumor vaccines are known to generate T_H_1-polarised CD4 + T cells to maintain and sustain anti-tumor CD8 + CTL responses. CD4 + T cells secrete IL-2 to directly activate CD8 + CTLs expressing the high-affinity IL-2 receptor and indirectly induce CD8 + CTL responses through the expression of co-stimulatory signals (B7 family ligands and IL-12) by up-regulating CD40 ligand to interact with CD40 on DCs. Tumor vaccines also boost CD4 + T cell-derived secretion of T_H_1-characteristic tumoricidal cytokines (e.g., IFNγ), which have direct anti-tumor activity. CD4 + T cell responses against tumors tend to be against self-derived epitopes [67]. Desialylation of glycan structures of tumor-associated antigens (self) and tumor-specific antigens (non-self) could facilitate MGL-mediated endocytosis and presentation by DCs [56]. The α2-3NA modified ID8 DWCTV with low sialic acid residues and high tumor antigenic Gal/GalNAc epitopes in our study induced Siglec E^low^/MGL^high^ DCs and the increased CD4/CD8 ratio in DLNs and the increased expression of IFNγ and IL-2 in peripheral plasma, indicating that vaccination with DWCTVs could activate both local and systemic immune responses.

Eliminating tumor cells by CD8 + T cells requires overcoming the immunosuppressive tumor environment. CD39 is a marker of exhausted tumor-infiltrating CD8 + T cells and co-expresses with multiple inhibitory receptors, such as PD-1, LAG-3, and Tim-3 [68, 69]. Siglec-9 is also reported to co-express with these inhibitory receptors [70] and potently attenuate the anti-tumor function of NK cells and T cells by binding sialoglycans in *trans* on OC cells [71]. The anti-tumor effects exhibited in *vivo* might demonstrate that ID8 DWCTVs desialylated to expose tumor antigenic Gal/GalNAc epitopes via hydrolyzing tumor cells with NAs could increase the immunogenicity of tumors [34, 72] and induce T-cell clones with TCR-Vβ repertoire diversity that overcome tumor- suppressive niches and kill tumor cells. Desialylation is a suitable treatment that contributes to the exposure of tumor-associated glycopeptide epitopes, which may be related to the immune recognition of tumor antigens on the surface of tumor cells [4, 73]. Thus, vaccination with DWCTVs seems to be a favorable way to elicit an anti-tumor immune response. More importantly, T cells within the tumor microenvironment and peripheral are abolished and irreversibly dysfunctional in tumor development, including reduced T cell activation and attenuated antigen-presenting cell responses. T cell-targeted interventions do not rescue T cell dysfunction marked by CD8 + CD39 + T cell clonal expansion, while tumor resection is sufficient to revert the systemic immune landscape [74, 75]. Therefore, immunization with tumor vaccines after tumor load reduction- a way to revive systemic immune function- is an ideal strategy to fight residual tumor cells and induce long-lasting anti-tumor immune responses to prevent disease recurrence [76].

## Conclusion

In summary, we report that DWCTVs with Gal/GalNAc epitopes deliver tumor antigens on the surface of tumor cells to the host immune system. Our findings highlight that DWCTVs could overcome sialoglycan-Siglec interaction-mediated immunosuppression and potentiate DC-mediated T cell anti-tumor immunity against OC in *vitro* and *vivo*. Thus, vaccination with DWCTVs would become an attractive intervention in improving the survival of OC patients after cytoreductive surgery.

## Supplementary Information

**Additional file 1: Fig. S1.** Screening the mitomycin C concentration for OC cell treatment. Representative images under phase contrast microscopy of OC cells treated with 0, 50, and 100 ng ml-1 mitomycin C for 2 h (**a**) and 3 h (**b**) and cultured for 24 h. Scale bars represent 100 μm.

**Additional file 2: Fig. S2.** Concentration-dependent curves of NA modification on OC cells. The α-2,3Sia (MAL II), α-2,6Sia (SNA), and Gal/GalNAc (PNA) epitopes expression on A2780 (**a**), OVCAR3 (**b**), and ID8 (**c**) cells were detected by FCM respectively after α2-3/α2-6NA modification. Plots represent three individual experiments; error bars are standard deviations (SD).

**Additional file 3: Fig. S3.** Representative confocal immunofluorescence images of OC cells. (**a**) and (**b**) showed the images of unmodified, α2-3NA modified, and α2-6NA modified ID8 cells stained by α-2,3Sia (MAL II, green), α-2,6Sia (SNA, green), and Gal/GalNAc (PNA, pink). Scale bars: 100 μm.

**Additional file 4: Fig. S4.** DCs maturation/activation and subsequently lymphocyte activation. The PBMCs (**a**), immature DCs (**b**), co-incubation of DCs and tumor cells (**c**), and co-culture of mature DCs and lymphocytes (**d**) were observed under a phase-contrast microscope. Scale bars: 25 μm.

**Additional file 5: Fig. S5.** Representative picture of HE staining for mouse ID8 OC. The section for tumors of nonvaccinated mice (**a**) and mice immunized with unmodified whole-cell ID8 vaccine (**b**), α2-3NA modified DWCTV (**c**), and α2-6NA modified DWCTV (**d**). Scale bars: 100 μm.

## Data Availability

All data generated or analysed during this study are included in this published article and its supplementary information files.

## Abbreviations

OC: Ovarian cancer
DC: Dendritic cell
Gal: Galactose
GalNAc: *N*-Acetylgalactosamine
Sia: Sialic acid
Siglec 9: Sialic acid-binding immunoglobulin-like lectins 9
MGL: Macrophage galactose type C lectin receptors
PD-1: Programmed cell death protein 1
Tim-3: T cell immunoglobulin and mucin-domain containing-3
LAG-3: Lymphocyte-activation gene 3
SHP 1: Src-homology domain 1
ZAP 70: Zeta-chain (TCR) associated protein kinase 70
TCR: T cell receptor
MHC: Major histocompatibility complex
FCM: Flow cytometry
IFNγ: Interferon γ
LDH: Lactate dehydrogenase
RT-qPCR: Real-time quantitative PCR
MAL II: Maackia Amurensis lectin II
PNA: Peanut agglutinin
SNA: Sambucus nigra lectin
PBMC: Peripheral blood mononuclear cell
IL 4: Interleukin 4
GM-CSF: Granulocyte–macrophage colony-stimulating factor
ELISA: Enzyme-linked immunosorbent assay
CD: Cluster of differentiation
AA: Amino acid
DLN: Draining lymph node
TIL: Tumor infiltrating lymphocyte
RNA: Ribonucleic acid
