# Supplementary figures and images for: Whole-cell tumor vaccines desialylated to uncover tumor antigenic Gal/GalNAc epitopes elicit anti-tumor immunity
Source: J Transl Med. 2022 Oct 31;20:496. doi: 10.1186/s12967-022-03714-y (PMC9620617; doi:10.1186/s12967-022-03714-y)

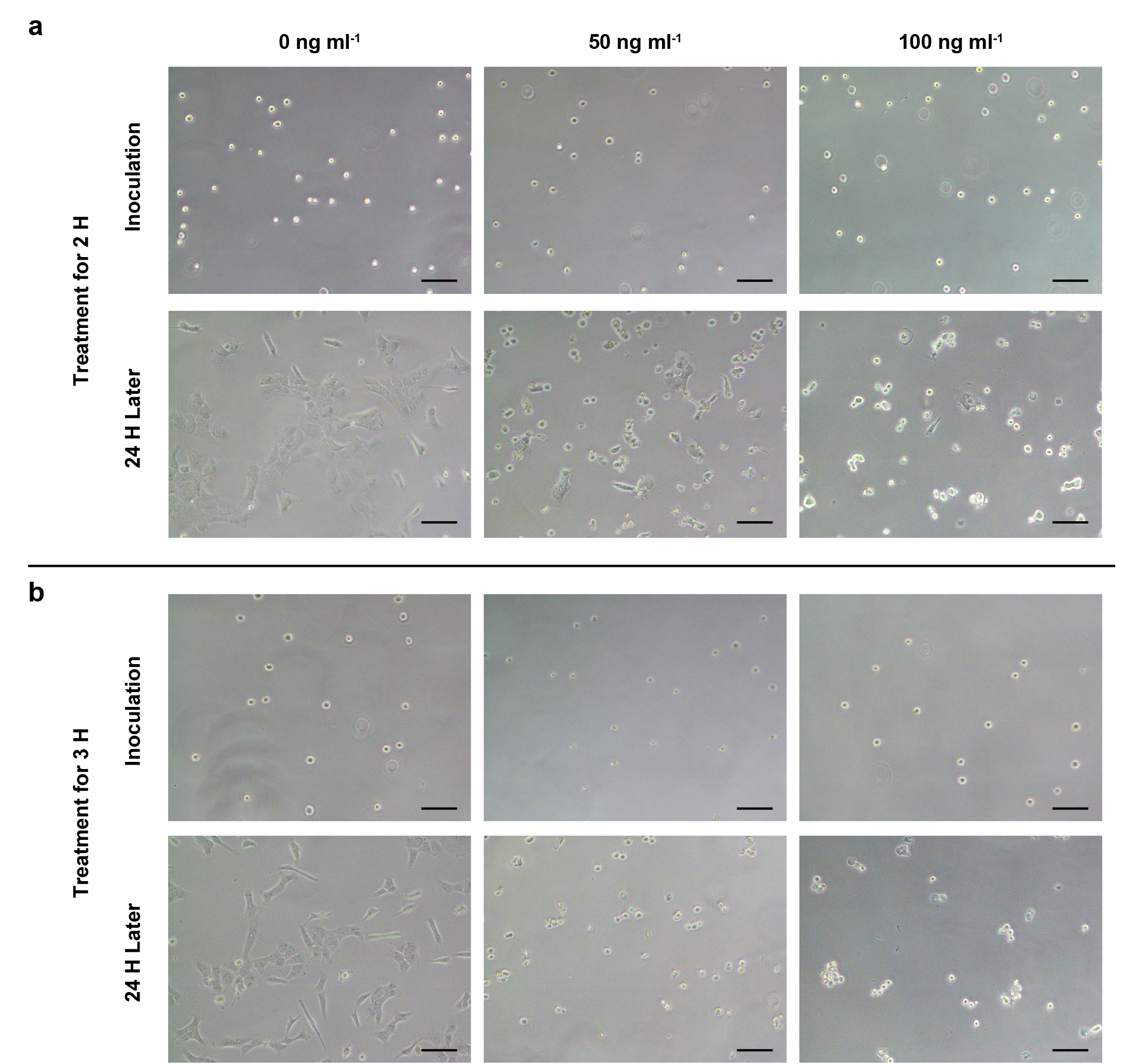

Supplement: Supplementary file 1 — Additional file 1: Fig. S1. Screening the mitomycin C concentration for OC cell treatment. Representative images under phase contrast microscopy of OC cells treated with 0, 50, and 100 ng ml-1 mitomycin C for 2 h (a) and 3 h (b) and cultured for 24 h. Scale bars represent 100 μm. [file 12967_2022_3714_MOESM1_ESM.png]

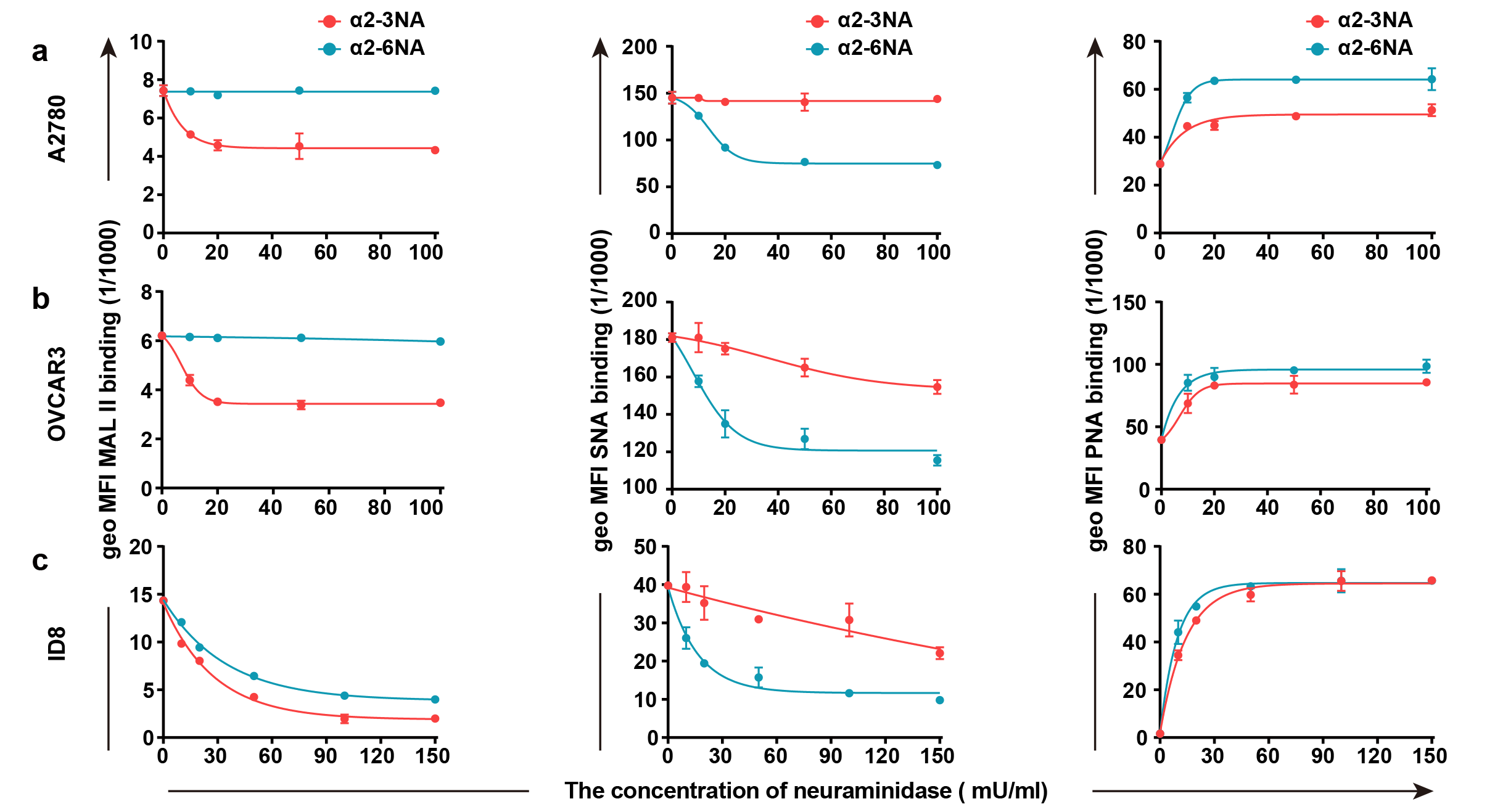

Supplement: Supplementary file 2 — Additional file 2: Fig. S2. Concentration-dependent curves of NA modification on OC cells. The α-2,3Sia (MAL II), α-2,6Sia (SNA), and Gal/GalNAc (PNA) epitopes expression on A2780 (a), OVCAR3 (b), and ID8 (c) cells were detected by FCM respectively after α2-3/α2-6NA modification. Plots represent three individual experiments; error bars are standard deviations (SD). [file 12967_2022_3714_MOESM2_ESM.png]

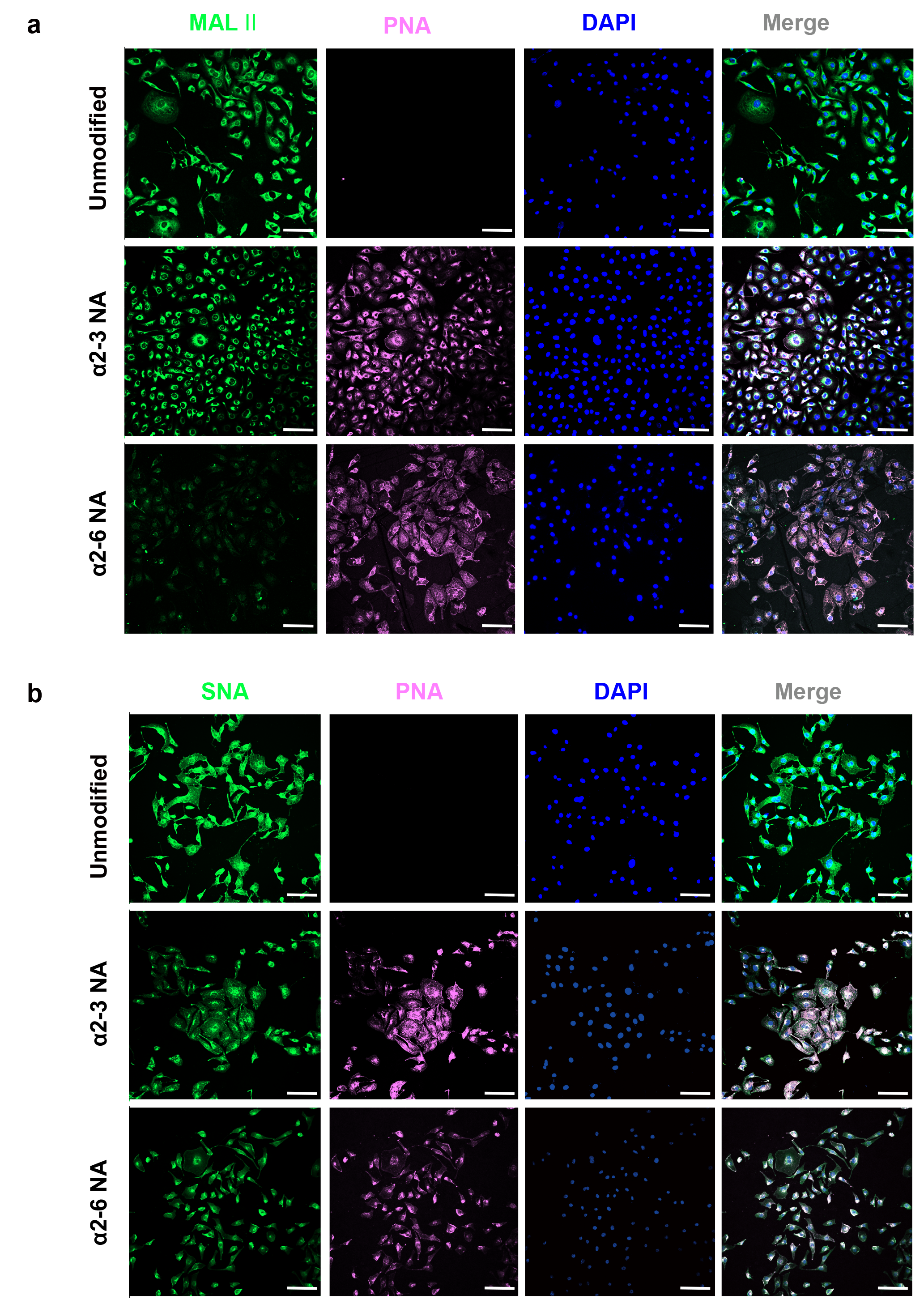

Supplement: Supplementary file 3 — Additional file 3: Fig. S3. Representative confocal immunofluorescence images of OC cells. (a) and (b) showed the images of unmodified, α2-3NA modified, and α2-6NA modified ID8 cells stained by α-2,3Sia (MAL II, green), α-2,6Sia (SNA, green), and Gal/GalNAc (PNA, pink). Scale bars: 100 μm. [file 12967_2022_3714_MOESM3_ESM.png]

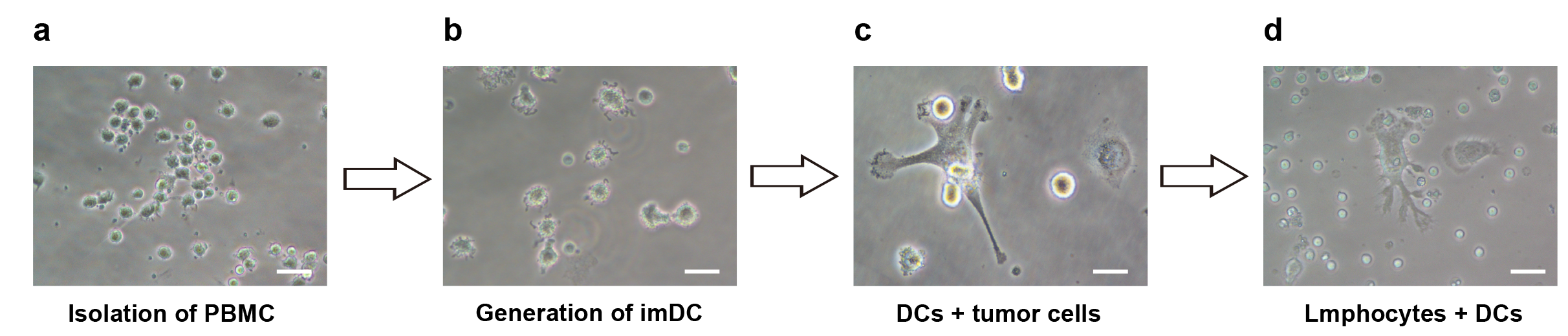

Supplement: Supplementary file 4 — Additional file 4: Fig. S4. DCs maturation/activation and subsequently lymphocyte activation. The PBMCs (a), immature DCs (b), co-incubation of DCs and tumor cells (c), and co-culture of mature DCs and lymphocytes (d) were observed under a phase-contrast microscope. Scale bars: 25 μm. [file 12967_2022_3714_MOESM4_ESM.png]

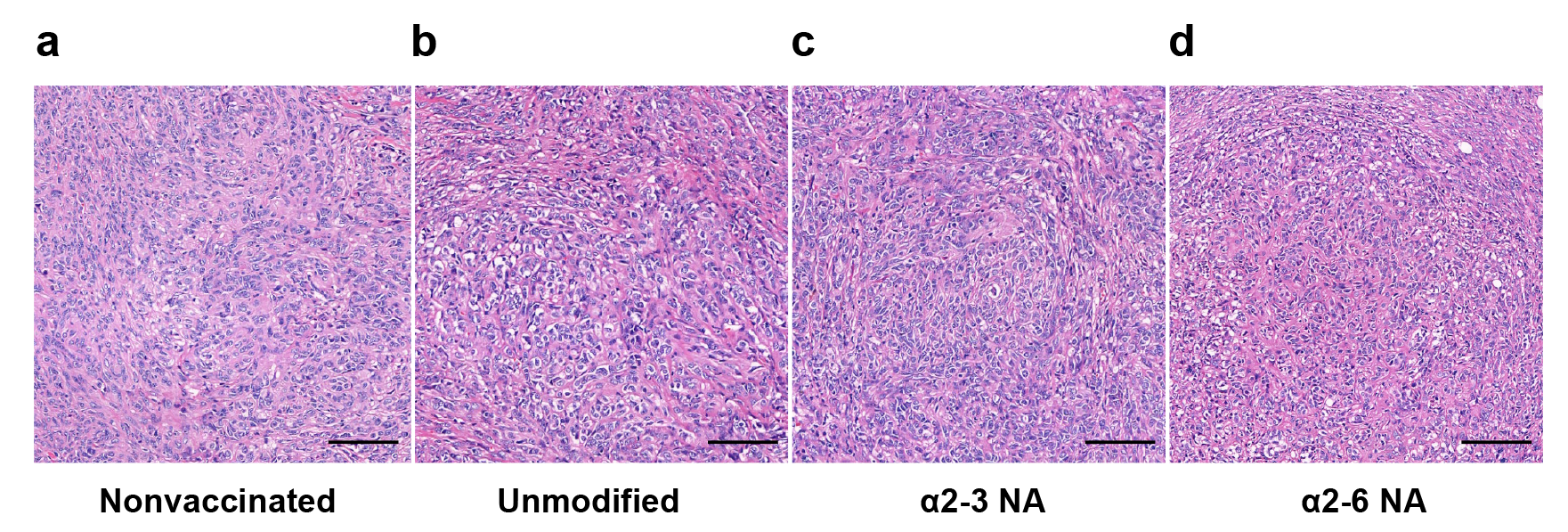

Supplement: Supplementary file 5 — Additional file 5: Fig. S5. Representative picture of HE staining for mouse ID8 OC. The section for tumors of nonvaccinated mice (a) and mice immunized with unmodified whole-cell ID8 vaccine (b), α2-3NA modified DWCTV (c), and α2-6NA modified DWCTV (d). Scale bars: 100 μm. [file 12967_2022_3714_MOESM5_ESM.png]
